# Diffusion in liquid mixtures

**DOI:** 10.1038/s41526-022-00246-z

**Published:** 2023-01-16

**Authors:** A. Vailati, H. Bataller, M. M. Bou-Ali, M. Carpineti, R. Cerbino, F. Croccolo, S. U. Egelhaaf, F. Giavazzi, C. Giraudet, G. Guevara-Carrion, D. Horváth, W. Köhler, A. Mialdun, J. Porter, K. Schwarzenberger, V. Shevtsova, A. De Wit

**Affiliations:** 1 Department of Physics “A. Pontremoli”, Università degli Studi di Milano, Milano, Italy; 2 E2S UPPA, CNRS, TotalEnergies, LFCR UMR 5150, Universite de Pau et des Pays de l’Adour, Anglet, France; 3 Mechanical and Industrial Manufacturing Department, Mondragon University, 20500 Arrasate-Mondragon, Spain; 4 Faculty of Physics, University of Vienna, Boltzmanngasse 5, Vienna, Austria; 5 Condensed Matter Physics Laboratory, Heinrich Heine University, 40225 Düsseldorf, Germany; 6 Department of Medical Biotechnology and Translational Medicine, Università degli Studi di Milano, Milano, Italy; 7 Thermodynamics and Process Engineering, University of Berlin, Ernst-Reuter-Platz 1, 10587 Berlin, Germany; 8 Department of Applied and Environmental Chemistry, University of Szeged, Rerrich Bela ter 1, 6723 Szeged, Hungary; 9 Physikalisches Institut, Universitat Bayreuth, 95440 Bayreuth, Germany; 10 MRC, CP165/62, Université libre de Bruxelles (ULB), av. F. D. Roosevelt, 50, B-1050 Brussels, Belgium; 11 Center for Computational Simulation, Escuela Técnica Superior de Ingeniería y del Espacio, Universidad Politécnica de Madrid, Plaza del Cardenal Cisneros 3, 28040 Madrid, Spain; 12 Institute of Fluid Dynamics, Helmholtz-Zentrum Dresden-Rossendorf, Bautzner Landstr. 400, 01328 Dresden, Germany; 13 Institute of Process Engineering and Environmental Technology, Technische Universität Dresden, 01062 Dresden, Germany; 14 Ikerbasque, Basque Foundation for Science, Bilbao, Spain; 15 Nonlinear Physical Chemistry Unit, Université Libre de Bruxelles (ULB), Campus Plaine, C.P. 231, 1050 Brussels, Belgium

**Keywords:** Chemical physics, Condensed-matter physics

## Abstract

The understanding of transport and mixing in fluids in the presence and in the absence of external fields and reactions represents a challenging topic of strategic relevance for space exploration. Indeed, mixing and transport of components in a fluid are especially important during long-term space missions where fuels, food and other materials, needed for the sustainability of long space travels, must be processed under microgravity conditions. So far, the processes of transport and mixing have been investigated mainly at the macroscopic and microscopic scale. Their investigation at the mesoscopic scale is becoming increasingly important for the understanding of mass transfer in confined systems, such as porous media, biological systems and microfluidic systems. Microgravity conditions will provide the opportunity to analyze the effect of external fields and reactions on optimizing mixing and transport in the absence of the convective flows induced by buoyancy on Earth. This would be of great practical applicative relevance to handle complex fluids under microgravity conditions for the processing of materials in space.

## Introduction

The diffusion of molecules is a fundamental mass transfer process where the random thermal motion of molecules in multi-component mixtures determines the relative motion of one species with respect to the others. The presence of external fields or of chemical reactions can significantly change the dynamics. A typical example is represented by a liquid mixture under the action of a macroscopic temperature gradient that determines a non-equilibrium mass flow of particles through the Soret effect. The presence of gravity can have a strong influence on the stability of a liquid mixture undergoing diffusion. A meaningful example is represented by convective instabilities driven by double diffusion, which can be triggered even when a liquid mixture exhibits an initially stable density profile. The interplay of the effect of gravity, of other external fields and of reactions can give rise to a complex stability diagram which shows that purely diffusive processes in multi-component mixtures can be hardly investigated on earth. For this reason, during the last 20 years, the European Space Agency has largely invested on the realization of Space experiments for the investigation of diffusion and thermodiffusion processes under microgravity conditions^1^. Within this context, the GRADFLEX project investigated heat and mass transfer diffusion processes at the mesoscopic scale on the FOTON M3 spaceship in 2007, providing convincing evidence that both processes are accompanied by large amplitude non-equilibrium fluctuations whose largest size coincides with that of the sample^2,3^. The IVIDIL project investigated the interplay between diffusion and vibrations under microgravity conditions on the International Space Station in 2009–2010, with the outcome that high-frequency periodic forcing generates time-averaged flows, which substantially affect the diffusive mass transfer^4^. The SCCO project involved many experiments performed on several space platforms, jointly supported by ESA and the China National Space Center, and by petrochemical industries including TOTAL, PETROCHINA and RIPED. The aim of the project was the investigation of thermodiffusion of multi-component mixtures under reservoir thermodynamic conditions of interest for the petroleum industry, with a focus on understanding how thermodiffusion affects the stability of the system^5–7^. The DCMIX project was aimed at the investigation of thermodiffusion in ternary liquid mixtures. Four space missions on the International Space Station, supported by ESA and Roscosmos, were performed from 2012 to 2018. The project allowed a systematic investigation of thermal diffusion in several model ternary mixtures, including mixtures selected to extend to ternaries the Fontainebleu benchmark previously developed for binaries^8^. The CHYPI project has, on the other hand, explored in microgravity chemo-hydrodynamic patterns due to the interplay between molecular diffusion and reactions^9–11,12^ .

In general, all these space projects were motivated by the fact that concentration gradients are associated with density gradients. As a result, any perturbation in the concentration of the mixture can either be stabilized by gravity or amplified by it, leading to the onset of convective instabilities.

## Key knowledge gaps

Mixing and transport of mass and/or heat in fluids is at the heart of numerous applications. We discuss a selection of them and explain the advantage of microgravity conditions in answering key questions in their respective fields.

### Confinement of fluctuations during diffusion processes

**Fig. 1:**
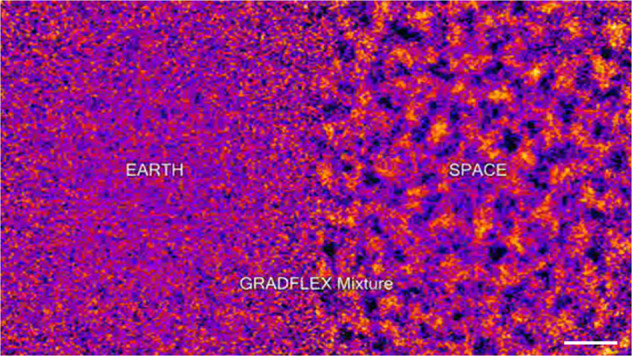
Non-equilibrium concentration fluctuations. False-colour shadowgraph images of non-equilibrium concentration fluctuations induced by the Soret effect in a polystyrene polymer solution on Earth (left) and in space (right) during the GRADFLEX experiment on FOTON M3^3^. Scale bar: 2.0 mm.

Mass diffusion and heat diffusion in liquid mixtures are triggered by composition and temperature gradients and are accompanied by long-ranged non-equilibrium fluctuations of concentration and temperature, respectively^13,14^. These fluctuations are limited in amplitude^15^ and lifetime^16^ by buoyancy forces. Gravity and diffusion are antagonist effects acting at different time scales that strongly depend on the fluctuation size. For small fluctuations, diffusion is the dominant mechanism while for large ones it is buoyancy. In the absence of gravity, fluctuations become giant—they grow proportional to the fourth power or their size—and they are limited only by the size of the sample container, as it has been demonstrated by the microgravity GRADFLEX experiment both for temperature and concentration fluctuations^2,3^ (Fig. 1).

The rigid boundaries influence large non-equilibrium fluctuations also in the presence of gravity. This confinement effect has been detected for concentration non-equilibrium fluctuations by dynamic-shadowgraphy and gives rise to a dramatic slowing-down of the dynamics of fluctuations^17^. For very thin samples, the confinement effect can be so large that the effect of buoyancy is totally cancelled out^18^. The forces generated on the fluctuations by the boundaries have a counterpart in a net force exerted on the boundaries themselves by the fluctuations. In fact, it has been predicted theoretically that the confinement of non-equilibrium fluctuations should lead to the development of non-equilibrium forces between the confining plates^19,20^. This phenomenon is similar to the critical Casimir effect, predicted in 1978 by Fisher and de Gennes^21^ and measured in 2008 by Hertlein et al.^22^, where equilibrium fluctuations become long-ranged close to the consolute critical point of a liquid mixture. Similar forces acting on colloidal particles dispersed in a critical mixture have been investigated under microgravity conditions in 2012^23^ by means of Near Field Scattering during the SODI-COLLOID experiment of ESA on the ISS.

In the case of non-equilibrium Casimir forces, the system does not need to be in the vicinity of critical conditions, but only out-of-thermodynamic equilibrium, a rather generic situation that is encountered in many practical cases of scientific or technological interest^19,20^.

The forces generated by the confinement of long-ranged fluctuations are present also between macromolecules thus altering their interactions, like it has been demonstrated in the case of critical Casimir forces. The experimental investigation of confined non-equilibrium fluctuations has both fundamental relevance for the understanding of interactions in non-equilibrium systems at the mesoscopic scale, a problem of quite general interest, and applicative relevance for microfluidic devices, Micro-ElectroMechanical Systems and biological systems. Experimental attempts made on Earth to detect these interactions have been hampered by the presence of buoyancy. As described above, on Earth the range of fluctuations is limited by the gravity force, which limits their amplitude and lifetime at small wave vectors^15^. These two aspects make the microgravity environment ideally suited for the investigation of forces generated by the confinement of fluctuations. The Giant Fluctuations (NEUF-DIX) and TechNES projects of ESA^24,25^ investigate non-equilibrium fluctuations in complex fluids under microgravity conditions and include experiments where non-equilibrium fluctuations are confined by colloidal particles dispersed in the sample fluid. From an experimental point of view, temperature gradients can readily be prescribed at the sample boundaries. Concentration gradients can be realized by preparing stratified layers of different compositions^16^, but a more elegant way is provided by the Soret effect^26–28^. This effect allows one to induce the composition gradient by a temperature gradient starting with a homogeneous equilibrium system that can be stored for almost arbitrary times prior to the actual experiment. Due to the time required to establish stationary convection-free non-equilibrium conditions, ideal Low Earth Orbit (LEO) platforms are represented by the ISS.

Confinement effects on the spatio-temporal behaviour of fluctuating fields are not restricted to a finite size of the sample container. Inside a porous medium, the average effective diffusion process is slowed down by the tortuous pathway of the solid matrix^29,30^. Recent experimental observations by heterodyne dynamic light scattering^31^ suggest that the dynamics of small fluctuations in density are dependent on the aspect ratio between the size of the fluctuation to the diameter of the pore.

### Non-equilibrium diffusion of particles in the presence and in the absence of external fields

**Fig. 2:**
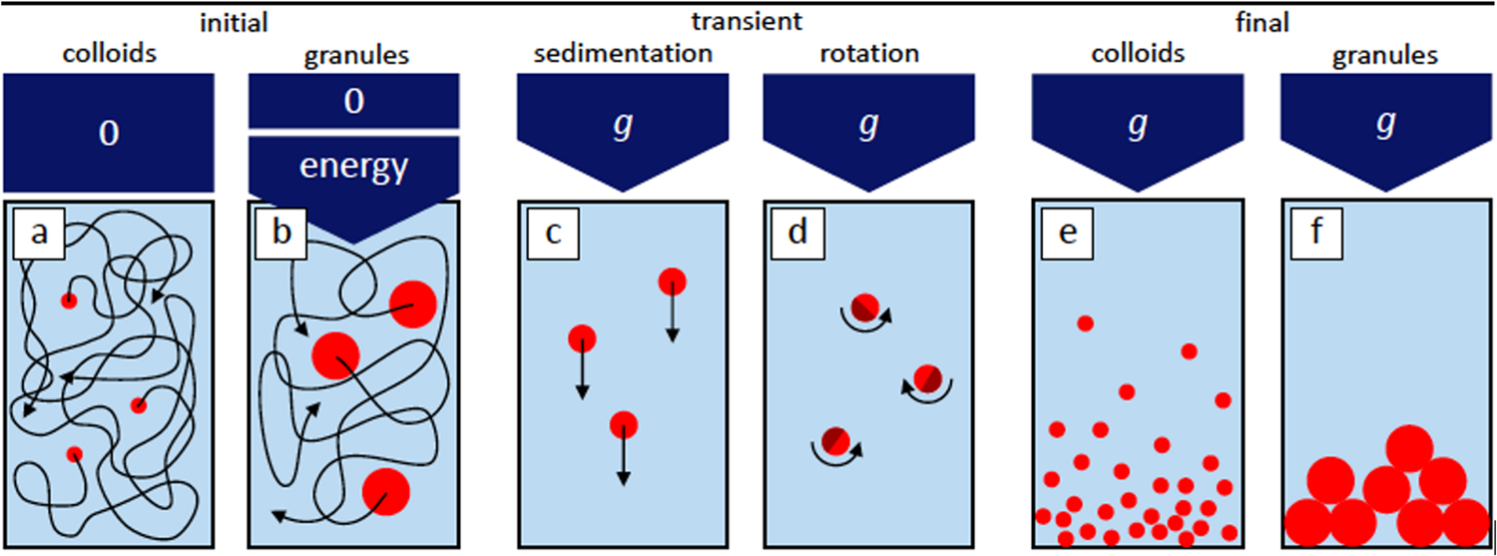
Effect of gravity on colloidal particles. Schematic representation of some key effects of gravity on small (colloidal) or large (granular) particles in a fluid. **a** In the absence of gravity, thermal fluctuations lead to a uniform distribution of colloidal particles. **b** For granular particles this process can be prohibitively slow, and hence injection of mechanical energy is required for the system to remain in a fluid state. **c** Gravity promotes a net flux of particles (sedimentation) along the direction of gravity. **d** If the mass distribution within each particle is not spherically symmetric, gravity results in a net torque on the particles, which can promote their orientational ordering. **e** For a diluted colloidal suspension, the steady-state distribution of particles in the presence of gravity is not homogeneous along the direction of gravity but corresponds to an exponential concentration profile. **f** In a granular medium, the particles densely accumulate at the bottom of the container, where they minimize their gravitational potential energy and form load-bearing frictional contacts.

Particles in a fluid respond to gravity in a way that critically depends on their size *a* and the density mismatch Δρ between particles and fluid (Fig. 2). This is captured by the Péclet number *Pe* = *a/l*_*g*_, where $l_g = k_BT/{{\Delta }}\rho V_pg$ is the sedimentation length, *k*_*B*_*T* is the thermal energy, *V*_*p*_ the volume of the particle and *g* the acceleration of gravity. Small colloidal particles, for which *Pe* << 1, and large granular ones, with *Pe* >> 1, behave differently: for colloids, the fluid acts as a thermal bath continuously exchanging energy with the particles and keeping them in perpetual Brownian motion; for granular systems, the effects of gravity dominate thermal agitation, rendering thermal fluctuations negligible. Accordingly, the granules sediment and accumulate at the bottom of the container (Fig. 2c, f). Inelastic collisions between granules result in energy distribution and frictional energy dissipation. This renders a continuous injection of energy necessary to maintain a fluid state^32–34^. This is achieved by, e.g. a gas or liquid flow or a mechanical or magnetic excitation, which, together with measurement facilities, has been realized for investigations in microgravity^35^ (Fig. 2b). Fluidized granular media represent an important model system to study mass and energy transport in athermal, intrinsically non-equilibrium conditions. Interestingly, after full equilibration, granules exhibit colloid-like features, including for instance density induced crystallization^36,37^, a diffusive-like particle dynamics in dilute and dense systems^33,35,38^. How similar colloids and fluidized granules are, is currently unclear. Microgravity experiments are an invaluable tool to avoid sedimentation, rendering an investigation of other factors easier.

Colloidal sedimentation is much better understood and investigated, starting with the seminal work of Jean Perrin^39^. Nevertheless, the presence of long-ranged hydrodynamic interactions still holds surprises: while theoretical models predict a divergence of the correlation length of velocity fluctuations in a sedimenting suspension, this divergence is not confirmed by experiments, leading to the so-called divergence paradox^40,41^. A possible solution may reside in the theoretical prediction that, during sedimentation, the fluctuations are quenched by gravity due to the coupling between the fluctuations and the concentration gradient^42^.

This quenching only occurs for length scales beyond a critical value, which may explain why large-scale fluctuations are not observed experimentally. The underlying theory^42^ also describes the occurrence of giant fluctuations during diffusion processes in molecular and macromolecular fluids^13,15^, whose properties have been extensively studied and confirmed also in microgravity during the GRADFLEX experiment^3,43,44^. More recently, giant fluctuations have also been confirmed in ground-based experiments with dilute^16^ and dense^45^ colloidal suspensions. However, the role of the coupling between fluctuations and concentration gradients for the divergence paradox needs to be clarified and requires experiments in which a homogeneous colloidal suspension is suddenly exposed to gravity and the spatio-temporal correlation of the fluctuations is measured as a function of time during the sedimentation. Such experiments are extremely challenging on ground because an artefact-free initial state is practically impossible to prepare. In contrast, microgravity platforms such as FLUMIAS, in which a microscope is coupled to a centrifuge, represent a unique opportunity: in microgravity a colloidal sample is left undisturbed to reach a homogenous state (Fig. 2a) and subsequently the centrifuge is started to induce sedimentation and the fluctuations are monitored (Fig. 2c) before an equilibrium concentration profile is attained (Fig. 2e). Varying the effective gravity between 0.01 and 1.1 g, the contribution of gravity can be singled out and hence the predictions^42^ tested.

The above examples focus on particles undergoing translational motion. Gravity can also induce rotation if a particle has an inhomogeneous or anisotropic density distribution (Fig. 2d). This includes particles used in self-assembly and some active Brownian particles, e.g. Janus particles. The effects of gravity^46^ are reduced in quasi-two-dimensional geometry. It is, however, desirable to extend experiments to three-dimensional situations^47^ for at least two reasons: first, to clarify the dependence of phenomena such as motility-induced phase separation on dimensionality^48^; second, to avoid direct or hydrodynamic interactions between the particles and the confining walls which might introduce spurious effects. Thus, microgravity experiments can offer opportunities for the investigation of non-equilibrium effects in a range of systems and various situations.

### Transport in multi-scale soft matter systems under external forces; interfacial dynamics and particle transport in forced fluid systems

Many soft matter systems of practical relevance exhibit a high level of complexity. There is a large knowledge gap in situations where different factors interact, like the simultaneous presence of small molecules, entangled polymers and large particles, in conjunction with an external temperature gradient or other external fields^49^. Transport mechanisms in multi-scale soft matter are not governed by a single “pure” effect, and an understanding of cross-interactions and their coupling is essential. These could include, for example, the interplay between mass and heat diffusion, viscoelasticity and volume flow. Complexity can originate not only from the number of constituents and driving forces but also from an intrinsic asymmetry of the components. A deep understanding of such complex dynamics could be achieved by starting with the investigation of low-concentration systems with one external driving force, increasing the complexity level towards crowded systems and biological aspects. The obtained results could allow the development of novel detection techniques for in situ sensing.

**Fig. 3:**
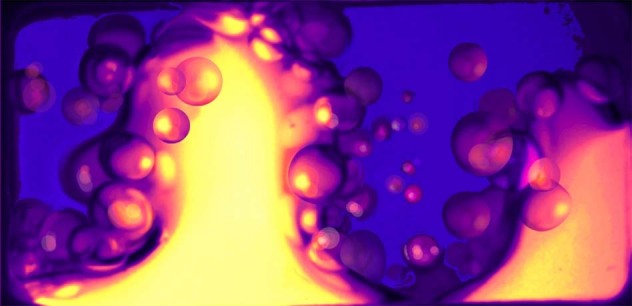
Interfacial dynamics of fluids in microgravity. Complex interfacial dynamics with FC-40 and 20 cSt silicone oil vibrated at 50 Hz in microgravity^50^.

The behaviour of interfaces^50^ plays an important role in forced multi-phase systems (Fig. 3), which arise in numerous applications in physics, engineering and biology. In particular, the capture of small particles dispersed in water (or brine) by viscoelastic fluids, such as mucus, is an issue of critical concern due to the growing problem of micro-plastic contamination in marine environments and their uptake by living organisms^51^. A systematic investigation of particle transport in the presence of more realistic flows, with time-dependent forcing representative of swimming, breathing through gills and wave motion, can be expected to provide a better understanding of the influence of fluid dynamics and interfacial phenomena on particle capture and to allow for more informed decisions about micro-plastics management and related problems such as filter design. Microgravity is particularly appealing for the analysis of inertial effects without the complications of buoyancy or sedimentation. Density matching (on ground) is undesirable, since it reduces inertial effects, and is impractical for realistic particle distributions. The exploration of interfacial dynamics and their role in particle uptake requires relatively long microgravity times (as on the ISS or Sounding Rockets) since the relevant phenomena develop and evolve slowly, depending on applied amplitude and frequency. With the low frequencies characteristic of swimming, breathing, or waves, sufficient particle capture events accumulate over many minutes. A better understanding of interface dynamics and particle transport has broad relevance for Human Space Exploration due to the importance of fluid management in life support and propulsion systems. Multi-phase systems also arise in numerous applications including resource extraction, which will be an important part of long-term Space Exploration.

### Diffusion of fluid mixtures across the Widom line—CO_2_ sequestration

Until a decade ago, the supercritical region was believed to be a featureless, homogeneous fluid-phase. However, contrary to popular belief, physically observable liquid- and gas-like subdomains can be found in the supercritical region^52^. The transition between these two subdomains occurs at the so-called Widom line, which can be considered as a continuation of the vapour pressure curve up to ten times its critical value^53^. There is still a large knowledge gap in the research field targeting diffusion of mixtures in the critical region and crossing the Widom line which requires significant development of experimental and theoretical approaches. In fact, although several industrial processes operate just above the critical point of the fluid, i.e. 1 < *T*/*T*_c_ < 1.1 and 1 < *p*/*p*_c_ < 1.5, where *T*_c_ and *p*_c_ are the critical temperature and pressure, respectively, reliable experimental data on mass diffusion, and particularly, on thermodiffusion of mixtures are scarce at these conditions^54^. An attempt to characterize the Widom line has also been reported by investigating the fluid density through the refractive index^55^.

The focus of this research theme lies on the quantification and understanding of the anomalies in mass- and thermal diffusion mechanisms related to the critical point and the Widom line in fluid mixtures. At the Widom line, thermophysical properties of fluids show an anomalous behaviour where a small temperature or pressure variation can lead to drastic changes of density, enthalpy, viscosity, etc. At the critical plait point theoretical studies predict singularities, i.e. a vanishing value of the mass diffusion coefficient as well as a diverging Soret coefficient and strong component segregation. Furthermore, the steady state may need an infinite amount of time to settle. An increase of the Soret coefficient with a critical slow-down of mass diffusion towards another type of criticality, i.e. the continuous line of consolute (demixing) points in ternary mixtures, has been observed in experiments on the ISS (DCMIX2 and DCMIX4)^1,56^. At the Widom line and near critical regions, fluid mixtures are very sensitive to any external perturbation, even small deviations from linearity of the temperature gradient may easily induce convection, which may disturb or erase the separation of components that is the hallmark of the Soret effect. In microgravity conditions, natural convection disappears in liquids even if a temperature gradient is applied. This creates an excellent possibility for the measurement and overall understanding of mass diffusion and thermodiffusion of fluid mixtures across the Widom line and approaching the critical plait point. Furthermore, high-quality convection-free thermodiffusion reference data are necessary for the development of theoretical models for the prediction of Fick diffusion and Soret coefficients of multi-component fluid mixtures.

**Fig. 4:**
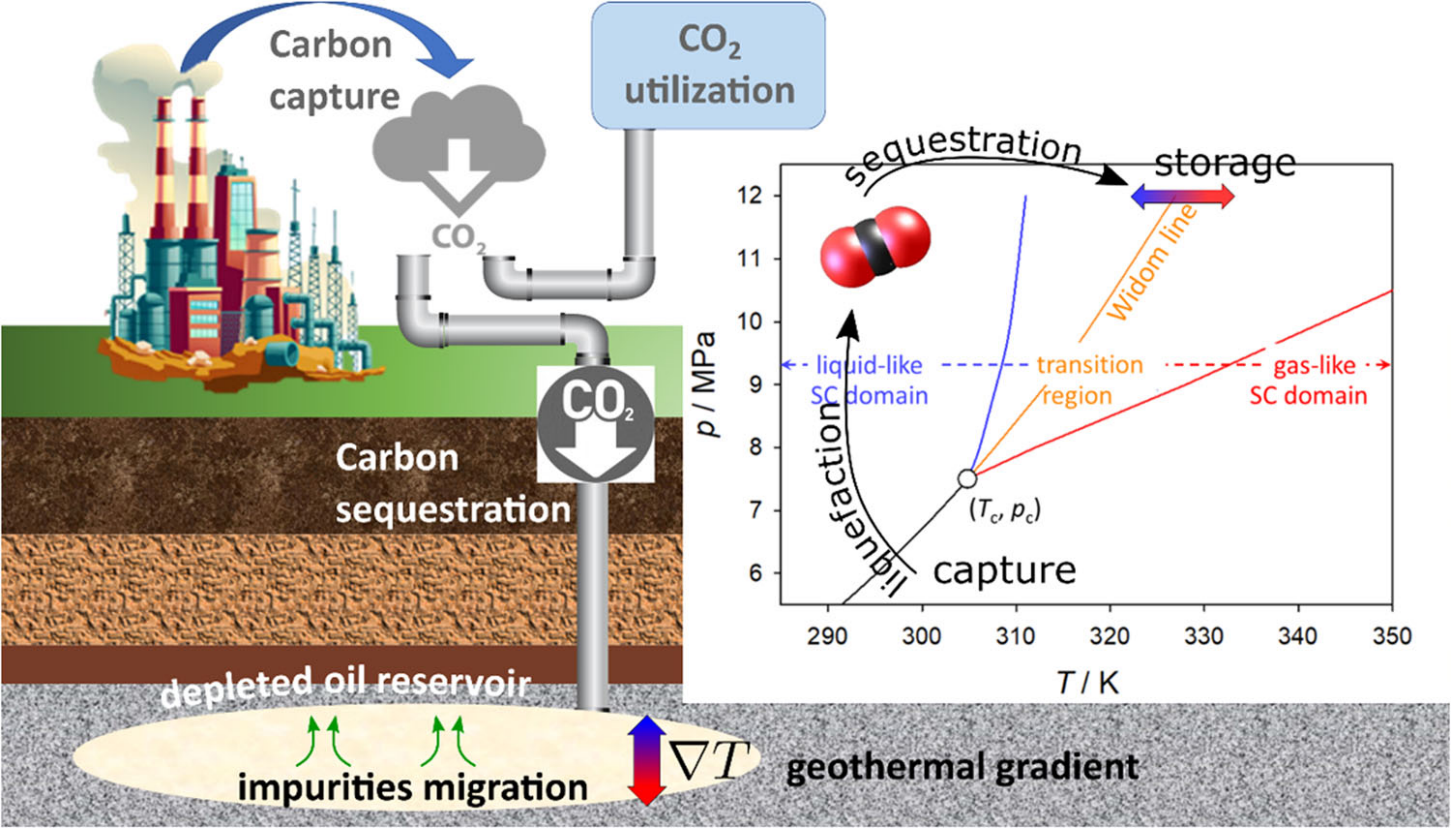
Carbon capture and sequestration. Schematic representation of carbon capture and sequestration in reservoirs. The inherent temperature gradient induces thermal diffusion of impurities from the bottom to the cap-rock of the reservoir. During injection, sequestration and storage, CO_2_ goes through different thermodynamic states as depicted in the right panel. The temperature and pressure dependence of the Widom line for pure CO_2_ was determined by the maxima of the isobaric heat capacity. The area between the blue and red lines represents the transition region between the liquid-like and gas-like subdomains of the supercritical fluid. Image of thermal-power plant by vectorpocket on Freepik.com (https://www.freepik.com/free-vector/heavy-industry-factory-working-thermal-power-plant-station-with-electricity_4758475.htm).

Of particular relevance is the investigation of carbon dioxide (CO_2_) mixtures in the context of carbon capture and sequestration. Indeed, one of the possible routes to reduce the level of CO_2_ in the atmosphere, following the GIEC recommendations^57^, is to capture CO_2_ at plants or directly from the air, and inject it into deep saline aquifers that are spread all over the world and specifically under the sea and can provide storage for multi-billion tons of CO_2_^58–60^. Deep saline aquifers are huge reservoirs made of porous rock with moderately low permeability sandwiched by layers of rock with very low permeability, the cap-rock, sealing the container especially on the top. In the early phase after injection, the near-well zone is particularly impacted with strong geochemical reactivity associated with intense mass and heat exchanges^61^. CO_2_ sequestrated is therefore subject to high temperature gradients and may be found in states across the Widom line and close to the critical point (Fig. 4). The unavoidable presence of impurities under these conditions may lead to a variety of non-equilibrium processes, each being important to perform a detailed quantitative reservoir risk assessment. For instance, mass- and thermodiffusion may result in the accumulation of chemically active species near the top of the cap-rock provoking CO_2_ leakage. A systematic investigation and understanding of the thermal transport of impurities in CO_2_ reservoirs can be expected to reduce the uncertainties related to risk assessment of CO_2_ geological storage.

The understanding of the dissolution process of CO_2_ into the brine is then of major relevance for the geological storage of large quantities of CO_2_. So far, the process has been largely investigated at the macroscopic scale to understand how convective motions on Earth could enhance the efficiency of the mixing process^62^. The progression and reactivity of CO_2_ at the microscopic level of the pores or at the mesoscopic level of the porous matrix still needs to be fully understood. In particular, the investigation of the role of fluctuations at the mesoscopic scale and of possible capillarity effects at the pore level would be of relevance for the understanding of real systems. The identification of pure Marangoni flows requires microgravity conditions to discriminate surface tension effects from buoyancy-driven progression of the CO_2_ in the host phase porous matrix, which contains both gas and liquid phases. Studies of convective dissolution of CO_2_ in model porous systems would therefore benefit from investigation in microgravity.

### Reactive flows

**Fig. 5:**
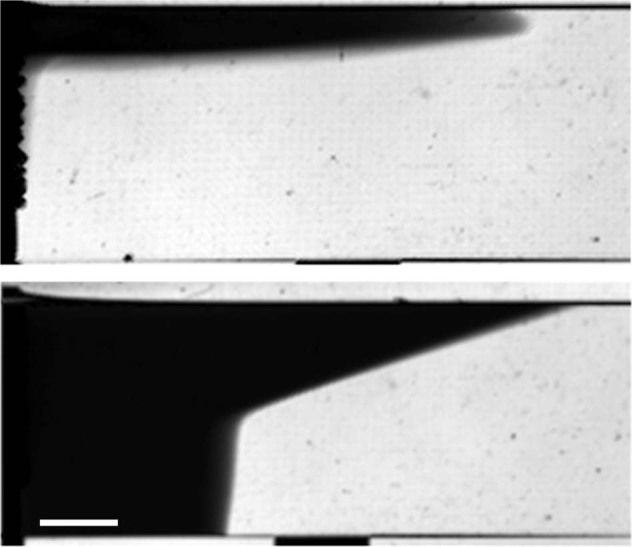
Marangoni-flow-driven chemical fronts. The front propagates from left to right in the presence of buoyancy (top) and in the absence of gravity (bottom). Scale bar: 5.0 mm.

In the presence of reactions, the dynamics can become even more complex because chemical reactions can change a physical property of the solutions at hand and thereby induce convective motions^63^. Gradients of composition can induce gradients of density, which are prone, on earth, to trigger buoyancy-driven currents. In the presence of an interface between two immiscible solvents, gradients in concentration can also initiate gradients of surface tension inducing Marangoni flows. On earth, it is challenging to disentangle buoyancy effects from Marangoni effects. Microgravity has played an important role in that respect, allowing to isolate Marangoni convection triggered by reactions along the interface between two immiscible solvents^64^. In such two-phase layers, the dynamics of A + B → C fronts, where reactant A diffuses out of one solvent into the other immiscible solvent containing reactant B, have been studied in parabolic flights^9^. It has been shown that a modulation of gravity induces acceleration/deceleration of the front and increases product formation^9^. Similarly, deformation of an autocatalytic front due to convective flows induced by self-sustained surface tension gradients could be analyzed in detail in sounding rocket flights thanks to the absence of buoyancy effects (Fig. 5)^10,11^.

**Fig. 6:**
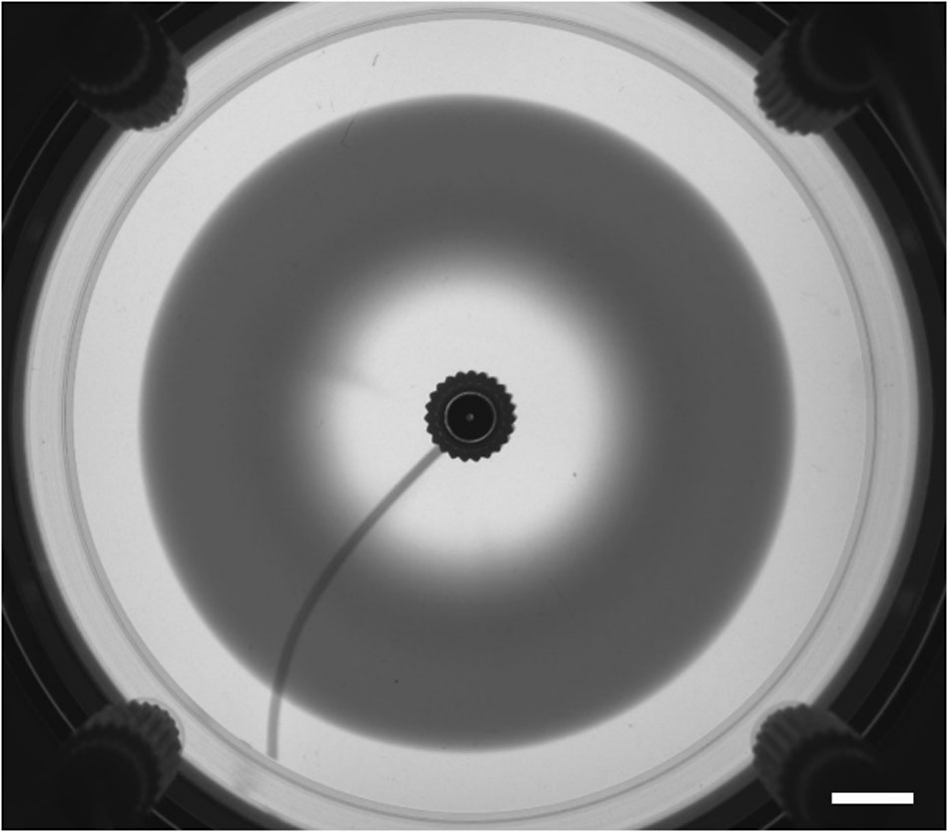
Reaction-diffusion-advection front in a chemical system. Radial reaction-diffusion-advection front generated by a simple bimolecular reaction A + B → C when a reactant A is injected at a constant flow rate in a quasi-2D reactor prefilled with the reactant B. In the absence of buoyancy-driven currents under microgravity, the dark product C spreads as an expanding circle^12^. Scale bar: 10 mm.

More recently, reactive fronts have been studied in flow-driven out-of-equilibrium conditions, which can be used to control the spatio-temporal distribution and amount of product C formed in time. Theoretical studies have shown that the temporal scaling of the properties of A + B → C fronts become then dependent on the injection rate^65^. On earth, testing these scalings is challenging because buoyancy-driven currents perturb the dynamics^66^. Microgravity conditions have here again allowed to test these scalings in absence of gravity (Fig. 6) and hence, have contributed to enlighten the influence that such density effects have on the behaviour of the front^12^.

A vast class of chemo-hydrodynamic patterns and instabilities remains, however, largely unexplored, namely the one involving a phase change like in precipitation fronts. In such systems, reactions locally generate a solid product, which may change the local density, viscosity or permeability of the medium. In addition, sedimentation can in some cases trigger a local clogging in confined reactors. Such dynamics have been recently studied for applications in CO_2_ sequestration techniques^67,68^, polymorph selection^69,70^, frontal polymerization^71^ or new material synthesis, to name a few. The specificity of precipitation reactions is that the solid product undergoes sedimentation and always changes the density of the solution, which naturally induces buoyancy-driven flows on earth. Disentangling such density-driven phenomena from other effects like viscosity or permeability related convective dynamics is crucial to optimize the processes but remains quite challenging^72^. Microgravity conditions will here play a crucial role in the future, providing substantial insights on the product properties, the yield of the reaction and the spatial distribution of the precipitate products. A further step advancing the knowledge on complex reactive flows includes precipitation processes in the vicinity of fluidic interfaces. The presence of the interface changes the nucleation of the precipitate particles, makes it necessary to consider Marangoni effects by changing reactant concentration, and can even lead to precipitate adsorption at the interface. These phenomena are strongly coupled to buoyancy effects so that a fundamental understanding can only be gained by microgravity experiments.

## Perspectives

The investigation of transport and mixing in fluids under microgravity is needed to achieve a deep fundamental understanding of the behaviour of multi-component fluids at the mesoscopic scale in the presence and in the absence of external fields. This kind of investigation will shed new light on the transport and mixing processes occurring in the Universe under various gravity conditions. A strong priority of the investigation of diffusion processes in mixtures is its relevance for Space Exploration. In fact, the sustainability of long-duration space travel will rely critically on the ability to process materials in the fluid state on site^73^, to mix fluids for the preparation of food, heating or cooling living chambers homogeneously, processing the CO_2_ released by the astronauts, etc. All these processes involve the transport and mixing of fluids such as gases or liquids under microgravity, or under gravity levels different from those of Earth. The processing will involve complex fluid materials such as fuels, food and construction materials. A suitable platform for these types of studies will be represented by the Deep Space Gateway, and by other platforms used to reach the Moon and Mars. We envision that after a conceptual phase, the development of suitable experiments will be driven by the practical experience collected during long-term missions, where complex fluids will be stored and processed under microgravity and reduced gravity conditions. Mixing processes may occur over a very wide range of time scales. This is because, under microgravity conditions, mixing processes do not exhibit a typical length scale, besides the microscopic size of the molecules and macroscopic size of the container where the mixing is taking place. For these reasons, diffusion in mixtures at the mesoscopic scale can be also investigated by performing experiments lasting for times as short as a few minutes, which can be executed on LEO facilities.

The general idea of the investigation of diffusion in mixtures is thus to understand the main factors which control the mixing of fluids in microgravity and, on this basis, to develop efficient approaches for the optimization of the transport of species and/or heat during mixing under various conditions relevant to space exploration.

## Benefit for Earth and industrial relevance

Besides space exploration, the understanding of transport and mixing in fluids is also a topic of great strategic and practical relevance for applications on earth. A huge variety of industrial processes are currently based on the processing of multi-component and multi-phase materials in a fluid state. Notable examples include the processing of foods, homogenization of blends in complex fluids like polymers, cements, metallic mixtures, as well as fluid control of chemical engineering processes. In porous media, transport is of tantamount importance in chemical engineering, oil recovery techniques, pollution remediation and the storage of CO_2_ into saline aquifers, to name a few examples. Depending on the goal, mixing between the displacing and the displaced fluids is either to be welcomed or avoided. Moreover, complex fluid materials structured at the mesoscopic scale are attracting increasing interest, due to the relevance of this range of length scales for living things. Of relevance are applications in the biomedical and pharmaceutical sectors, and reducing the pollution of micro-plastics. On Earth, mixing between reactive or non-reactive fluids of different composition and/or at different temperatures often involves various processes including buoyancy-driven flows. Microgravity conditions can help to isolate the other effects and allow a better understanding of their role in the control and efficiency of mixing and transport.

## Outlook and summary

The investigation of the diffusive mass transport in liquid mixtures in the past and present years has largely benefitted from the opportunity of performing experiments under reduced gravity conditions by using sounding rockets and Low Earth Orbit platforms, such as the International Space Station. The reduced gravity conditions allowed to perform experiments under purely diffusive conditions, in the absence of convective instabilities driven by the gravitational acceleration, and to investigate the interplay between diffusion and chemical reactions, between vibrations and diffusion, to perform accurate measurements of the transport coefficients of multi-component mixtures, and to elucidate the role of fluctuations at the mesosocopic scale during diffusion processes. Although microgravity experiments are still important for a fundamental understanding of the diffusive mass transfer in complex fluids, the significance of these studies is gradually shifting towards the understanding of the behaviour of multi-component mixtures under hypergravity and reduced gravity conditions. This topic is of great relevance for long-duration space exploration missions, where the ability of processing complex liquids represents a strategic habilitating factor.

### Reporting summary

Further information on research design is available in the Nature Research Reporting Summary linked to this article.

## Supplementary information

Reporting Summary
